# A targeted screening method for non-invasive vascular assessment of the lower limb

**DOI:** 10.1186/s13047-016-0181-2

**Published:** 2016-12-07

**Authors:** Peta Ellen Tehan, Vivienne Helaine Chuter

**Affiliations:** School of Health Sciences, Faculty of Health, University of Newcastle, Ourimbah, NSW 2258 Australia

**Keywords:** Non-invasive vascular assessment, Podiatrist

## Abstract

**Background:**

Podiatrists routinely perform non-invasive lower limb vascular assessment, however frequently cite time as a major barrier in performing regular assessment. The aim of this study was to develop an evidence-based vascular assessment method to guide podiatrists’ decision-making processes to aid in timely vascular assessment in at risk populations.

**Method:**

The sample underwent brachial pressure measurement, ankle pressures, toe pressure and Doppler waveform with colour duplex ultrasound (CFDU) used as the reference standard. Both the targeted screening method and the American Heart Association (AHA) guideline for vascular screening were then applied to the data set and sensitivity and specificity of each method was calculated.

**Results:**

One hundred nineteen participants were included. Sensitivity of the targeted screening method (62%, 95% CI 47.17–75.35) was higher than the AHA method (49%, 95% CI 34.75–63.40), however, specificity of the AHA method (94%, 95% CI 85.62–98.37) was higher than the targeted screening method (85%, 95% CI 74.26–92.60). Diagnostic accuracy was similar with the AHA method yielding 74% diagnostic accuracy and the targeted screening method 73%.

**Conclusion:**

The targeted screening method and the broad international guideline demonstrated similar accuracy, however clinicians may save time using the targeted screening method. This study highlights the difficulties in obtaining accuracy in lower limb vascular assessment in general.

## Background

Identifying the presence and extent of peripheral arterial disease (PAD) through accurate lower limb vascular assessment is essential for reducing morbidity and mortality associated with the disease [1]. Through early identification of PAD, complications such as ulceration, gangrene and amputation can be reduced or avoided using aggressive risk factor modification, provision of ongoing foot care and foot care education [2–4]. It has been estimated that up to 90% of amputations are preventable [2–4] with adequate foot screening including vascular assessment playing a vital role in reducing complications and improving clinical outcomes [1]. Accurate and effective vascular assessment requires a complex reasoning process which takes into account a patient’s vascular risk factors as well as an awareness of the effect of co-morbidities on the clinical efficacy of assessments techniques, and, subsequent interpretation of results to formulate an evidence-based management plan.

Podiatrists play a central role in conducting non-invasive lower limb vascular assessments in the general population. We have recently demonstrated that on average, podiatrists perform two vascular assessments per day, however, the type of the testing that is conducted during the assessments is extremely varied and, potentially inadequate for accurate PAD screening [2]. There are several available international guidelines for performing screening for PAD, including National Institute of Health and Care Excellence (NICE) guidelines and the American Heart Association (AHA) guidelines. Both of these guidelines recommend the use of ABI as a primary screening tool for populations at risk of PAD [3, 4]. However the uptake of these recommendations into clinical practice appears to be inconsistent [2]. Time required to perform recommended objective testing, particularly the ankle-brachial index (ABI) is the most widely nominated barrier to conducting appropriate vascular assessment, [2, 5] with clinicians often relying on more quickly applied assessments including continuous wave Doppler (CWD) and pulse palpation. In addition there is growing evidence of the reduced accuracy of the ABI for detecting PAD in specific populations including those at risk of medial arterial calcification (MAC), particularly when co-existing with PAD and of a more distal distribution of atherosclerotic lesions including diabetes, renal disease, and older aged cohorts [6]. In such populations further alternate testing including the toe brachial index (TBI) is frequently required, adding to the time to complete an assessment. Our recent research suggests more quickly applied vascular assessment techniques such as the TBI and CWD may be suitable for use as first line assessment techniques for PAD assessment, particularly in older people and those with diabetes [7, 8]. The aim of this study was to determine if a targeted version of current guidelines in which the TBI was used initially in patient populations in which the ABI is known to be problematic could achieve similar diagnostic accuracy to testing protocols where the ABI is used as the primary objective testing method for all people at risk of PAD.

## Methods

The CFDU and vascular assessment results of this data set has previously been used to investigate diagnostic accuracy of vascular testing methods in specific populations [7, 8]. Participants were recruited on a volunteer basis from two different locations, a community health centre in Newcastle, NSW, and a private podiatry practice in Nelson Bay NSW. Participants who fitted the AHA guidelines for peripheral vascular screening were eligible to participate; i.e. patients over the age of 65, patients above the age of 50 with the presence of diabetes or currently smoking or patients with exertional leg pain. Participants who were unable to comply with the testing protocol or who had a vasospastic disorder preventing TBI measurement were excluded. Testers included three vascular sonographers who performed: colour duplex ultrasound (CFDU), ankle, brachial and toe pressure measurements, at a private clinic in Newcastle. Methodology for vascular screening has previously been described [7, 8]. CFDU reliability has previously been assessed [8] and found to be acceptable.

**Fig. 1:**
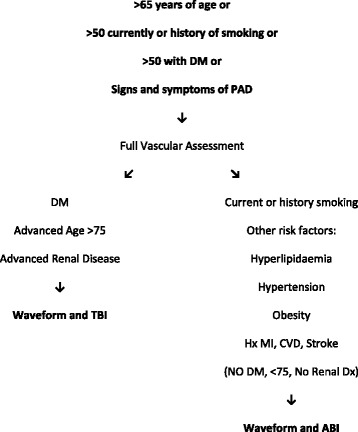
Targeted screening method for non-invasive vascular assessment of the lower limb

**Fig. 2:**
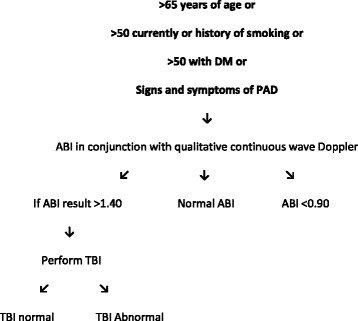
American Heart Association guideline for non-invasive peripheral vascular screening

An extensive review of the literature was performed. Combined with recent research completed by the researchers [7, 8] which examined the diagnostic accuracy of the ABI, TBI and CWD in different populations at risk of PAD, a targeted vascular assessment method was developed that is applied based on a patient’s medical history (Fig. 1). The targeted method used the patient’s risk factors for PAD combined with the known limitations of the ABI to assist the clinician choose the most accurate vascular test in the specific patient population being assessed. In the targeted method the presence of diabetes and/or renal disease, or being of advanced age were used as a prompt for the clinician to perform a TBI due to the reduced diagnostic accuracy of the ABI in these populations [6, 7, 9]. In the targeted method all other risk factors for PAD led the clinician to perform an ABI as this has been demonstrated to be an adequate test in the general population at risk of PAD and, in the absence of diabetes, renal disease or advanced age [10]. All patients had CWD performed as this is an accessible, quick and relatively simple test to perform which has been shown to be reliable and accurate in populations requiring vascular screening and a useful adjunct to peripheral pressure testing [7, 9, 11, 12]. The targeted method was then directly compared to the American Heart Association (AHA) guideline (Fig. 2) [3] to determine relative diagnostic accuracy of both screening techniques for PAD. Ethics approval was obtained through the University of Newcastle ethics committee and Hunter New England Area Health research ethics committee.

### Experimental procedure

The AHA guideline was applied to the entire data set by a single researcher (PT) i.e. the ABI result was used unless it exceeded 1.4 in which case it was replaced by the TBI. These results were used to determine the diagnostic accuracy of the AHA guidelines for detecting PAD using CFDU as the reference standard. The targeted method was also applied to the entire data set by a single researcher (PT) i.e. the ABI was used unless diabetes or renal failure was present or participants were aged over 75 years in which case the TBI value was used. These results were used to determine the diagnostic accuracy of the targeted screening method for detecting PAD using CFDU as a reference standard.

For statistical calculations relating to diagnostic accuracy, presence of PAD was defined as one or more arteries with >50% stenosis [13, 14]. Sensitivity, specificity, positive and negative predictive values and likelihood ratios were calculated with 95% confidence intervals for the AHA screening method and the targeted screening method. Calculations of diagnostic accuracy were performed using Microsoft Excel.

## Results

A total of 120 participants were recruited (Table 1) however one participant was excluded as the CFDU scan was performed on a different day to the remainder of the vascular examination. An additional two participants were excluded from the targeted screening method due to missing toe pressure data. Generally the population was older, in accordance with the inclusion criteria. There were a high number of participants with diabetes (61%). Sensitivity of the targeted method (62%, 95% CI 47.17–75.35) was higher than the AHA method (49%, 95% CI 34.75–63.40), however specificity of the AHA method (94%, 95% CI 85.62–98.37) was higher than the targeted screening method (85%, 95% CI 74.26–92.60) (Table 2). Overall the diagnostic accuracy of both methods were similar, with the AHA screening method 74% diagnostic accuracy and the targeted screening method 73% diagnostic accuracy.

**Table 1:** Participant information

Total participants (N)	119
Males n (%)	75 (63.02)
Females n (%)	44 (36.97)
Age Range (Years)	53–92
Diabetes n (%)	73 (61.34)
Mean Age (years)	73.1 (SD^A^ 7.2)
Incompressible ankle pressure n (%)	16 (13.44)
Distal PAD n (%)	37 (31.09)
Proximal PAD n (%)	7 (5.88)
Distal & Proximal PAD n (%)	7 (5.88)
PAD n (%)	51 (42.85)
Proximal Occlusions n (%)	1 (0.84)
Distal Occlusions n (%)	40 (33.61)

^A^ = standard deviation, *PAD* Peripheral arterial disease

**Table 2:** Results

Results table				
Participant group	Targeted screening method		AHA	
	%	95% confidence interval	%	95% confidence interval
Sensitivity	62.00	47.17 to 75.35	49.02	34.75 to 63.40
Specificity	85.07	74.26 to 92.60	94.12	85.62 to 98.37
Positive predictive value	75.61	2.25 to 7.66	86.21	68.34 to 96.11
Negative Predictive Value	75.00	0.31 to 0.65	71.11	60.60 to 80.18
Positive likelihood ratio	4.15^b^	2.25 to 7.66	8.33^a^	3.09 to 22.45
Negative likelihood ratio	0.45^b^	0.31 to 0.65	0.54	0.41 to 0.71
Diagnostic Accuracy	73.94		74.78	

^a^Important likelihood ratio ^b^May be important likelihood ratio

## Discussion

This study investigated whether diagnostic accuracy of lower limb vascular screening for PAD can be achieved using a targeted version of current guidelines designed to reduce the time taken to perform a vascular assessment. The results of this study indicate that the targeted method had a higher sensitivity for PAD than when tests were conducted in accordance with the AHA guidelines, however lower specificity. Overall the two methods had almost identical diagnostic accuracy (AHA method 74%, targeted method 73%). Although the ABI has been shown to have good sensitivity and excellent specificity across the general population [10] our recent research suggests uptake of the test by podiatrists is poor, with the time associated with performing the test cited as one of the most common reasons for this [2]. Performing an ABI requires two ankle pressures per limb (dorsalis pedis and posterior tibial). The targeted method we have proposed increases the number of people who have a TBI performed as the initial screening test. A TBI is quicker to perform due to the need for only one toe pressure per limb to be taken. In addition the targeted method ensures there will rarely be a time that clinicians need to perform more than one form of lower limb pressure measurement in a single testing session. Both changes are likely to reduce the amount of time needed to perform objective non-invasive vascular testing.

Current evidence suggests podiatrists rely on subjective findings including pulse palpation and visual appearance to identify PAD, while objective assessment is often limited to CWD which we have shown to have poor reliability [2, 15]. The method we have developed offers a potential mechanism to improve the diagnostic accuracy of vascular assessments currently performed by podiatrists by targeting the type of objective test to be used using patient medical history. In addition, increasing the use of the TBI, which has been shown to have high reliability in diabetes and non diabetes cohorts for initial testing for PAD [16], offers a more time efficient objective test that may be more widely adopted in clinical practice. There is also growing evidence that tests such as the TBI may be a valuable adjunct to clinical practice and could be more widely used, particularly in populations at risk of foot complications [17].

Of note our study demonstrates that neither screening method yielded a very high level of diagnostic accuracy, which re-enforces the difficulty of non-invasive lower limb vascular assessment in populations at risk of PAD. Further investigation into the diagnostic accuracy of non-invasive vascular assessment testing methods should be undertaken to ascertain what testing should be performed in populations at risk of PAD. The diagnostic accuracy of both the ABI and TBI should be further elucidated using gold standard imaging as a reference standard. Further research that helps guide clinical practice could facilitate increased efficiency and increased accuracy when conducting vascular assessments, reducing the number of undiagnosed cases of PAD and ensuring timely intervention and appropriate management to prevent complications such as ulceration and infection and amputation.

The results of this study need to be considered in light of some significant limitations. The accuracy of both screening tools relies upon the individual accuracy of each diagnostic test. Each of the included tests, ABI and TBI have their own limitations with accuracy. The ABI in particular has been shown to have limited diagnostic accuracy in populations at risk of PAD. The reference standard used, CFDU, whilst a valid form of diagnostic imaging, and used extensively clinically, also has limitations with diagnostic accuracy. Ideally angiography should be used as a reference standard however due to the prospective nature of the data collection for this study this was not possible. Future research should use retrospective data and use the gold standard in vascular imaging, angiography as a reference standard.

## Conclusion

Modification of current international guidelines based on medical history to reduce the time burden of lower limb vascular assessment in clinical practice yields similar diagnostic accuracy to assessment undertaken in accordance with the guidelines. This study highlights the difficulties in obtaining accuracy in lower limb vascular assessment in at risk populations and clinicians should consider using the TBI as an alternate screening tool given its higher level of accuracy and predictive capabilities.
